# Enhancing on/off ratio of a dielectric-loaded plasmonic logic gate with an amplitude modulator

**DOI:** 10.1038/s41598-023-30823-5

**Published:** 2023-03-28

**Authors:** Kai-Hao Chang, Zhan-Hong Lin, Po-Tsung Lee, Jer-Shing Huang

**Affiliations:** 1 Department of Photonics, College of Electrical and Computer Engineering, National Yang Min Chiao Tung University, Hsinchu, 300 Taiwan; 2 Leibniz Institute of Photonic Technology, Albert-Einstein Straße 9, 07745 Jena, Germany; 3 Institute of Physical Chemistry and Abbe Center of Photonics, Friedrich-Schiller-Universität Jena, Helmholtzweg 4, 07743 Jena, Germany; 4 Research Center for Applied Sciences, Academia Sinica, Taipei, 11529 Taiwan; 5 Department of Electrophysics, National Yang Ming Chiao Tung University, Hsinchu, 30010 Taiwan

**Keywords:** Nanophotonics and plasmonics, Photonic devices

## Abstract

Plasmonic waveguides allow focusing, guiding, and manipulating light at the nanoscale and promise the miniaturization of functional optical nanocircuits. Dielectric-loaded plasmonic (DLP) waveguides and logic gates have drawn attention because of their relatively low loss, easy fabrication, and good compatibility with gain and active tunable materials. However, the rather low on/off ratio of DLP logic gates remains the main challenge. Here, we introduce an amplitude modulator and theoretically demonstrate an enhanced on/off ratio of a DLP logic gate for XNOR operation. Multimode interference (MMI) in DLP waveguide is precisely calculated for the design of the logic gate. Multiplexing and power splitting at arbitrary multimode numbers have been theoretically analyzed with respect to the size of the amplitude modulator. An enhanced on/off ratio of 11.26 dB has been achieved. The proposed amplitude modulator can also be used to optimize the performance of other logic gates or MMI-based plasmonic functional devices.

## Introduction

Using photons for communication and circuit operation features lower loss, higher frequency, large bandwidth, and thus faster speed compared to conventional electronics. Modern photonic technology has been advanced to overcome the limitations of conventional electronics, such as interconnect delays and increased power dissipation in electronic transistors^1,2^. However, the footprint of photonic circuits using dielectric waveguides is rather large due to the cutoff of photonic modes. Moreover, the performance in term of the on/off ratio is relatively low compared to electronic circuits. These drawbacks limit the miniaturization and applications of photonic devices. Merging electrons and photons, surface plasmon polaritons (SPPs) offer unique opportunities for next-generation optical nanocircuits and communication^3–5^. The advantages of using SPPs as information carriers lie in the ultra-high operating frequency in the optical range and cutoff-free field confinement down to the nanoscale^6–9^. Based on these characteristics, plasmonic nanocircuits and waveguides are considered as potential substitutes for dielectric photonic devices, in particular for matching nanoscale electronic devices for optoelectronic modulation^10^. Related plasmonic optoelectronic components, such as modulators^11–14^, filters^15–17^, switches^18–21^, circuits^8,22–25^, sensors^26^, have been demonstrated and reported.

One of the main drawbacks of plasmonic waveguides compared to photonic waveguides is the rather challenging fabrication, high losses, and limited propagation length. To address these issues, well-designed dielectric materials have been used in combination with plasmonic substrates^27^. These DLP devices offer the possibility to prolong the propagation length to millimeter range^28–31^ and allow easy design and fabrication of subwavelength optical devices^32–34^. In addition, combining dielectric materials with plasmonic substrate also allows the introduction of gain materials for loss compensation or even laser^35–37^, as well as phase-changing materials for active control^38–42^. For subwavelength waveguide and circuit operation in the optical regime, DLP waveguides are promising for their relatively easy fabrication, low loss, and potential of interconnection with silicon photonic circuits^43^. Low-loss power splitters and spatial light modulators based on DLP devices have been demonstrated at telecommunication frequencies^44^. Broadband operation with high transmittance has been demonstrated with a footprint as small as several hundreds of nanometers^45,46^. In addition, DLP waveguides also provide a convenient platform for light coupling to optical fibers and other silicon-on-insulator devices fabricated by complementary metal-oxide semiconductor-compatible processes^47^.

To perform logic operation and other essential functions of a microprocessor on DLP logic gates, the most widely used approach is to introduce a well-designed patch of DLP waveguide that properly accommodates MMI^48^, which generates predictable and fruitful spatial distribution of power flux due to the constructive and destructive interference between fundamental and high-order plasmonic modes. This approach avoids the use of Mach–Zehnder-based architecture and thus reduces the loss^49,50^. Based on the MMI scheme, Fukuda et al*.* have purposed the DLP devices for half and full adder operation, based on a 1 × 1 MMI phase modulators and a 2 × 2 MMI intensity modulators^51,52^. The contrast ratios of the AND and XOR operations of their adders reach 5.82 and 17.16 dB, respectively, as measured by scanning near-field optical microscopy. The measured near-field interference patterns require careful analysis because of the inevitable contribution of photonic modes in the thick dielectric layer. The main challenge is the loss due to the complex connections between logic gates and the relatively poor logical performance, i.e., low on/off ratio, compared to typical metal–oxide–semiconductor field-effect transistors. Although many functions have been proposed and demonstrated, weak logic performance due to low on/off ratio remains the main challenge for plasmonic logic gates to enter the realm of practical applications. For the same reason, developing and fabricating useful logic conjunctions, which are critical for AND and XNOR logic operations, remains extremely difficult. Only a few previous related works have been reported but the corresponding on/off ratios are rather low^53–55^.

To improve the on/off ratio of DLP waveguide-based logic gates, we introduce an amplitude modulator for the first time in this work. MMI scheme is employed to design the compact DLP waveguide-based XNOR logic gate. We theoretically demonstrate that the amplitude modulator helps achieve complete constructive and destructive interference. We demonstrate a high contrast ratio of XNOR logic operation by using a specific arrangement of amplitude and phase modulators. The proposed amplitude modulator is not limited to the XNOR gate shown in this work but also applies to all MMI-based DLP functional devices, which requires high on/off ratio.

## Results

**Figure 1:**
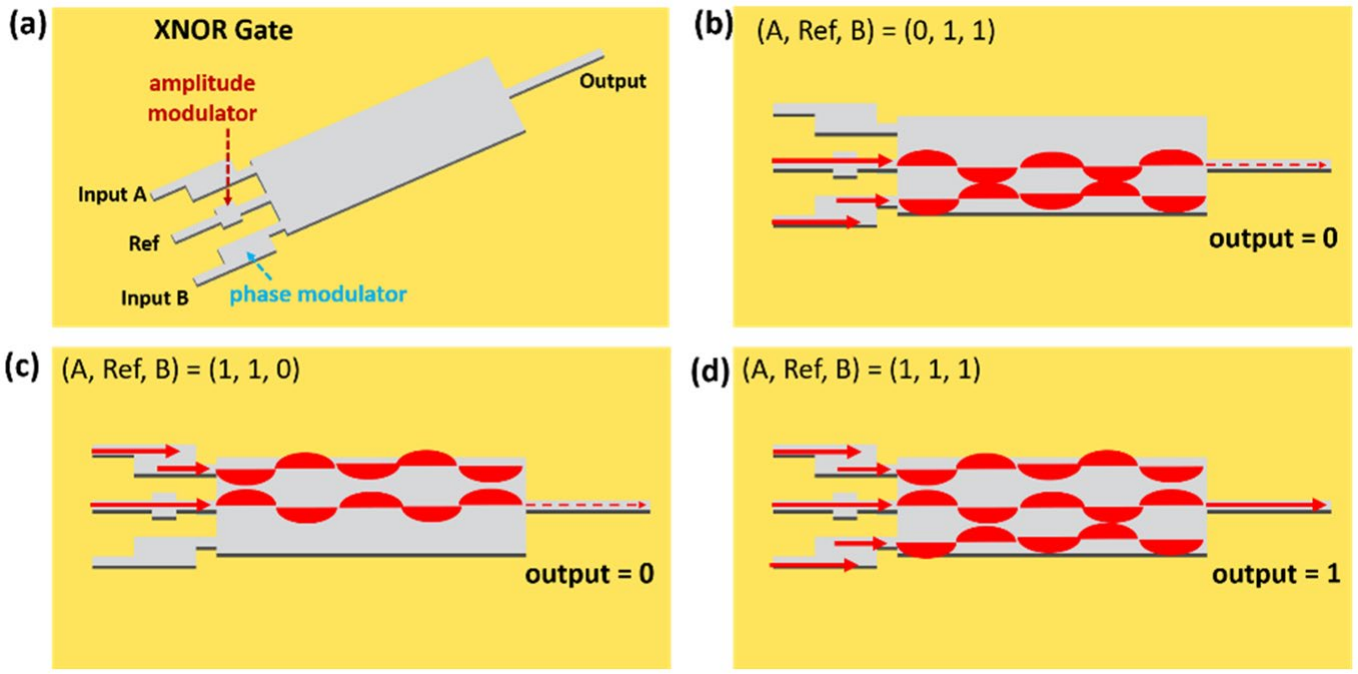
(**a**) The schematic diagram of plasmonic XNOR gate. Input A and input B contain a phase modulator. The Ref channel has an amplitude modulator. (**b**,**c**) The cases of zero output when (A, Ref, B) = (0, 1, 1) and (1, 1, 0). (**d**) The case of unitary output when (A, Ref, B) = (1, 1, 1).

The schematic diagram of the plasmonic XNOR gate used in this work is shown in Fig. 1a. It comprises two input waveguides (input A and input B) and one reference waveguide (Ref). Each of the two inputs is equipped with a phase modulator and the reference waveguide has an amplitude modulator. Three waveguides inject SPPs modes into a common plate for MMI. The designed logic operation for off and on states is illustrated in Fig. 1b–d. The “off” state occurs when either input A or input B interferes with the mode from the reference to produce destructive interference at the output channel (Fig. 1b,c). As for the “on” state, it occurs when both input A and input B inject the modes to interfere with the mode from the Ref channel. This leads to a significantly large power transmission through the output channel (Fig. 1d).

**Figure 2:**
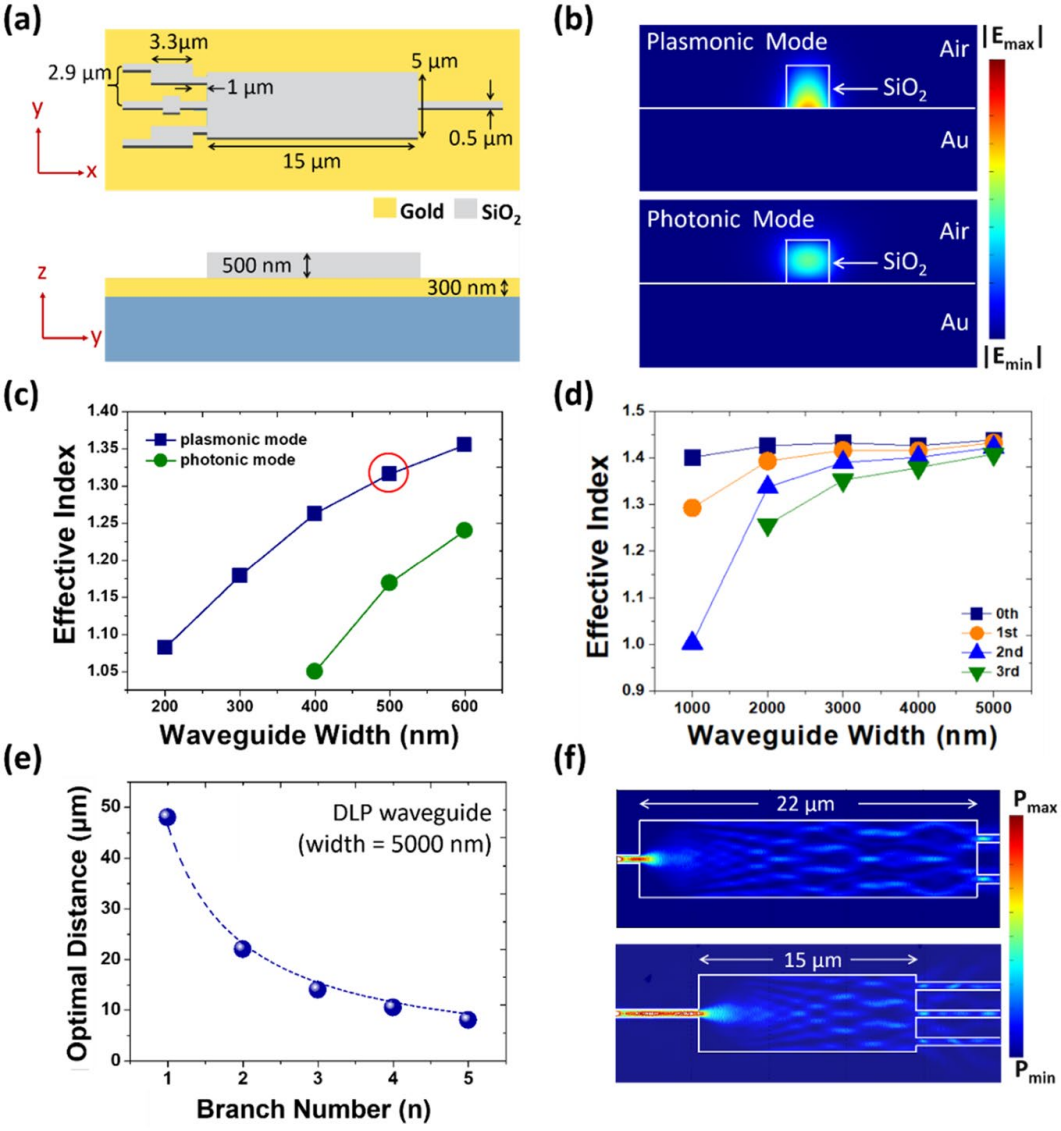
(**a**) Dimension of the plasmonic XNOR gate in the x–y (top panel) and y–z plane (bottom panel). Gold and SiO_2_ are chosen as the material for the substrate and the dielectric waveguide, respectively. (**b**) Simulated cross-sectional electric field mode profile of the fundamental plasmonic (top panel) and photonic (bottom panel) guided mode in a 500 × 500 nm^2^ SiO_2_ dielectric channel on a gold substrate. White solid lines mark the boundaries of the dielectric waveguide and the surface of the gold substrate. (**c**) Simulated effective indices of the fundamental plasmonic (navy blue squares) and photonic mode (dark green dots) as functions of the width of the SiO_2_ dielectric waveguide between 200 and 600 nm. (**d**) Same plot as (**c**) with only the indices of fundamental and high-order plasmonic guided modes with respect to the waveguide width over the extended range of 1000–5000 nm. Apart from the fundamental plasmonic mode (navy blue squares), the first-order (orange dots), second-order (blue triangles), and third-order (green inverted triangles) plasmonic modes can also exist in the DLP waveguide. (**e**) The optimal distance for n-branched power splitting plotted versus the number of branches, n. Dots are data points obtained from COMSOL simulation and the dashed curve is obtained from fitting the simulated data with Eq. (1). (**f**) Simulated spatial distribution of optical power flow for the cases of two- (top panel) and three-branched power splitting (bottom panel) cases. The input on the left side of the image is a single SiO_2_ channel (cross-section: 500 × 500 nm^2^) on a gold substrate and the following MMI section has the same height but a much larger width of 5000 nm with lengths optimized for two- and three-branched splitting.

The in-plane and cross-sectional dimensions of the designed XNOR gate used in the simulation are shown in Fig. 2a. We have chosen SiO_2_ as the dielectric material because it is compatible with modern CMOS fabrication process. We note that DLP waveguides with a sufficiently large cross section of the dielectric channel can support both the plasmonic and the photonic modes. The cross section of our dielectric input channels is as large as 500 × 500 nm^2^. Therefore, it also supports both the plasmonic and the photonic modes. Nevertheless, the plasmonic mode can be readily distinguished from the photonic mode by examining the mode profile. The plasmonic mode features a spatial distribution that exponentially decays from the metal/dielectric interface, whereas the photonic mode exhibits a mode distribution with maximum intensity around the center and decays toward the boundary of the dielectric channel. Figure 2b shows the simulated mode profiles of the fundamental plasmonic (top panel) and photonic mode (bottom panel) of our DLP waveguide. The effective index and mode order of the guided plasmonic mode vary as a function of the waveguide width. As shown in Fig. 2c, fundamental plasmonic mode (the 0th order at a vacuum wavelength of 785 nm) emerges at the waveguide width of 200 nm, which is much smaller than the case of fundamental photonic mode. The effective index of the plasmonic guided mode increases with increasing the waveguide width and saturates at around 1.40. With relatively small cross section, the guided fundamental plasmonic mode features high mode index and absence of cutoff.

Further increasing the width of the dielectric channel to more than 1000 nm allows guiding the high-order transverse plasmonic modes (Fig. 2d). The effective index of all modes eventually converges to around 1.40, which is determined by the index of the dielectric material and the index of the metal substrate. Based on the results of mode analyses, we have chosen 500 nm as the width of the input and Ref ports because this width supports a sufficiently high effective index of the fundamental plasmonic mode without the introduction of high-order plasmonic modes. As for the section for MMI, we have chosen a width of 5000 nm for two reasons. First, this width is sufficient to accommodate all the input and output channels. Secondly, DLP waveguide of this width also supports high orders of plasmonic modes. This is critical for the MMI as will be discussed later. The length of the MMI section is calculated to be 15 μm according to the following formula^56,57^,

1 $$L = \frac{P}{N}\left( {L_{\pi } } \right),$$

where *P* denotes the period along the propagating direction and ${L}_{\pi }$ denotes the beating length of the two lowest-order modes, which can be expressed as

2 $$L_{\pi } \approx \frac{\pi }{{\beta_{0} - \beta_{v} }} = \frac{{4n_{eff} W^{2} }}{{3\lambda_{0} }}.$$

${\beta }_{o}$ and ${\beta }_{v}$ are propagation the constants in air (n = 1) and in the DLP waveguide, respectively. The latter depends on the modal profile and is indicated by the mode number *v.* For *v* = 1, ${\beta }_{v}$ matches the fundamental transverse standing wave condition. *W* and ${n}_{eff}$ are the width of DLP waveguide and the effective refractive index of the guided mode, respectively. ${\lambda }_{o}$ = 785 nm is the operating wavelength in air. Along the propagating direction, mode beating results in evolving transverse field profile, which can be described by the mode transition number n. Equations (1) and (2) can be used to analytically calculate the optimal distance required for the transverse mode profile to transform into a specific n-fold intensity distribution. This calculation is important for the design of DLP logic gates because the calculated optimal distance determines the optimal length of the MMI section for targeted n-branched power splitting. In principle, these formulas allow us to determine the length of the MMI waveguide section in order to split the power into any number of branches. We verify Eq. (1) with electromagnetic simulations using COMSOL. Figure 2e plots the optimal distances for the corresponding branch number n obtained from COMSOL simulation, which can be well fitted with Eq. (1). The simulated spatial distributions of the guided power in MMI sections designed for two- and three-branched power splitting are given as examples in Fig. 2f. With the input of a fundamental plasmonic mode from a single dielectric channel (cross-section: 500 × 500 nm^2^) on a gold substrate, the DLP waveguide sections of lengths 22 and 15 μm serve as interfering plates for MMI, leading to optimized one-to-two and one-to-three power splitting, respectively.

**Figure 3:**
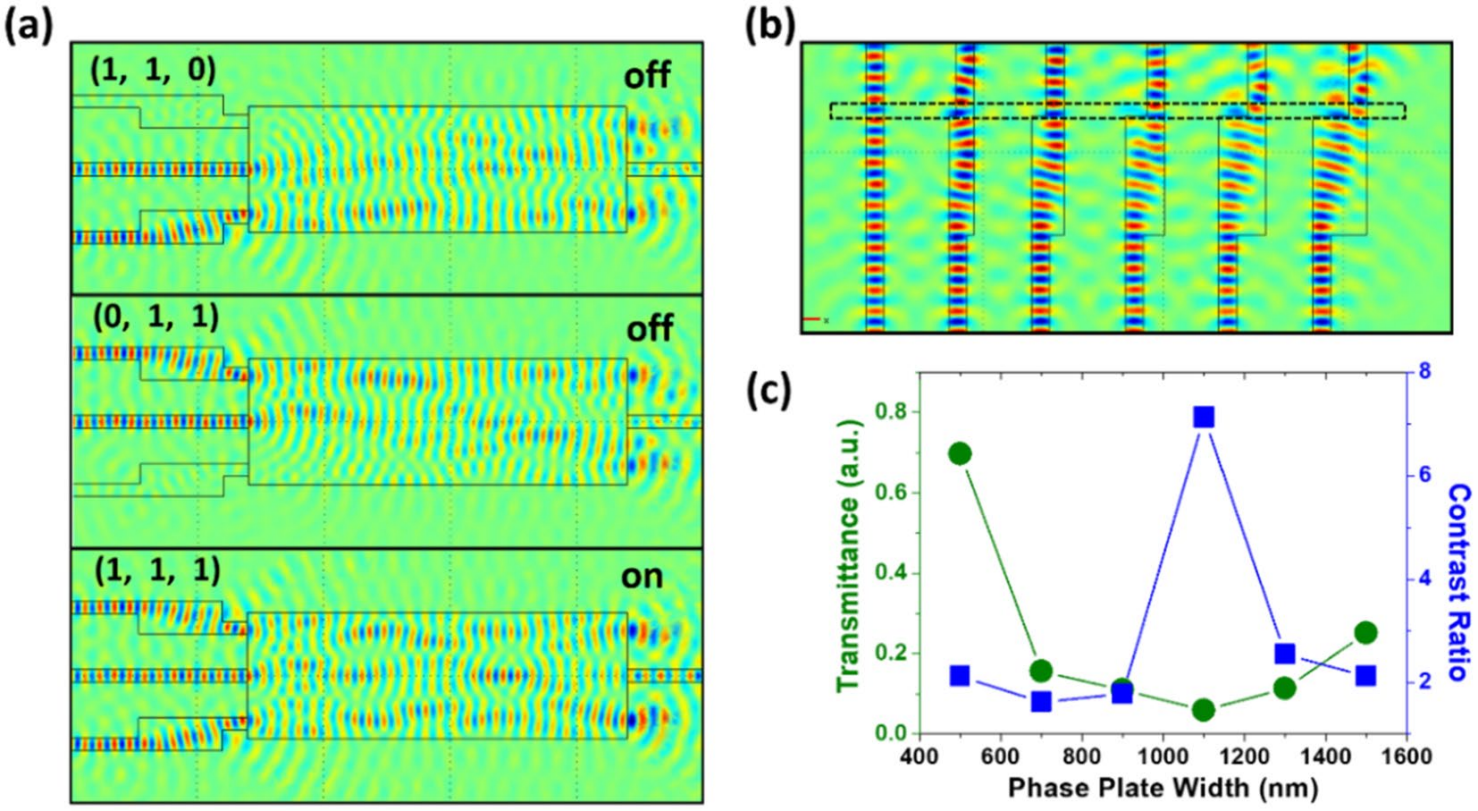
(**a**) Simulated electric field (E_z_) of the XNOR gate with phase modulators in the waveguides of ﻿input A and input B. Boolean operations of “off” (top and middle panels) and “on” states (bottom panel) at different input conditions. “Off” and “on” states correspond to very low and high power transmission through the output port on the right. (**b**) Simulated electric field (E_z_) distributions revealing the phase modulation at different widths of the phase tuning plate. (**c**) The transmittance and contrast ratio of the XNOR gate as a function of the width of the phase modulator. The contrast ratio is defined as the power of the “on” state over that of the “off” state.

Having analyzed the important parameters for designing DLP logic gates, we further demonstrate that the logic operation of the XNOR logic gate shown in Fig. 2a can be enhanced by introducing well-designed phase and amplitude modulators. We first demonstrate an XNOR logic gate with only a phase modulator but without any amplitude modulator at A and B input ports. The simulated E_z_-field distribution in Fig. 3a shows the logic performance of the XNOR gate. Various input conditions of the XNOR logic operation lead to the “on” and “off” states, showing distinctively different E_z_-field intensity at the output port on the right side. The Ref port plays an important role in the logic operation of an XNOR gate. Destructive interference for the “off” state comes from the superimposition of the input signals. Since the dimensions of the DLP waveguide section for MMI are fixed, it is important to optimize the phase difference between the input modes for perfect destructive interference. For this, the modulation of the input phase has been achieved by controlling the width of the phase modulators, which are the wider plates in input A and input B. To optimize the logic operation, we have scanned the width of the phase modulator at a fixed length for both input ports. Figure 3b shows the simulated real electric field in the DLP logic gate. The phase of the guided mode shifts as a function of the width of the phase plate. Increasing the width allows adjusting the phase of the electric field up to π, seen as the color change from blue to red in the area indicated by black dash lines. The phase variation induced by the modulator can be analytically described as^51,56^

3 $$\Delta \varphi_{a} = \left( {\beta_{s} - \beta_{a} } \right)L_{a} \approx \left( {\frac{1}{{W_{se}^{2} }} - \frac{1}{{W_{ae}^{2} }}} \right)\frac{{\pi \lambda_{0} }}{{4n_{eff} }}L_{a} ,$$

where ${\beta }_{s}$ and ${\beta }_{a}$ are the propagation coefficients of the fundamental mode in the single waveguide and the phase modulator, respectively. *W*_se_ and *W*_ae_ are the effective waveguide widths for the fundamental mode in the single-mode waveguide and phase adjuster, respectively. ${L}_{a}$ is the length of the phase modulator. We adapt this design of phase plate to the XNOR gate.

Figure 3c shows the transmittance and contrast ratio of the XNOR gate by with respect to the width of the phase plate. Here, the contrast ratio is defined as the output power of the “on” state over that of the “off” state. The transmittance is obtained by dividing the output power by the sum of the power of all inputs. For our XNOR gate at “off” state, the minimum transmittance and maximum contrast ratio are obtained at 1100 nm. Low transmittance with enhanced contrast ratio is the optimization target for the phase modulator in an XNOR gate at “off” state.

**Figure 4:**
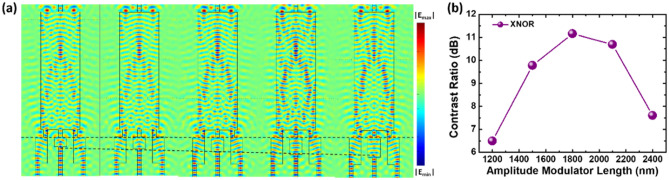
(**a**) E_z_-field distribution of the XNOR gate in the “on” state. The length of the amplitude modulator on the Ref port is varied as indicated by the black dashed lines. (**b**) Relation of contrast ratio as the function of amplitude modulator length.

To further improve the performance of the logic gate, the amplitude should also be optimized. For this purpose, we introduce an amplitude modulator to the Ref input port with the best optimized phase modulators on ﻿input A and input B. Figure 4a shows the E_z_-field distribution of the XNOR gate obtained with an amplitude modulator at different lengths installed on the Ref port. By optimizing the length of the amplitude modulator on the Ref port, the on/off ratio of the logic performance can be enhanced up to 11.26 dB at the length of 1800 nm. This is higher than the 8.57 dB (ratio = 7.2) of the original design. As shown in Fig. 4b, by modulating the optical loss in the amplitude modulator, near-perfect destructive interference in the “off” state can be achieved within a limited range of modulator length (approximately 1500–2300 nm), resulting in an improved contrast ratio.

In this work, we focus on the theoretical demonstration of the amplitude modulator. Here, we briefly discuss the fabrication possibilities and errors. In the simulations, the thickness of the dielectric layer has been set to a constant value. In real applications, the designed structures can be realized by modern nanofabrication technologies, such as electron-beam lithography, focused ion beam milling, nanoimprint, or nanoprinting methods. The imperfection of the nanofabrication may, however, lead to a discrepancy between the theoretical prediction and the performance of a real structure. We briefly discuss the possible fabrication errors and their impacts. Since the designed structures do not contain sub-50 nm features, such as nanogaps, the above-mentioned modern nanofabrication technologies should be sufficient in terms of spatial resolution. However, keeping a constant thickness of the dielectric layer might be challenging. Uncertainty in the thickness of the dielectric layer may lead to a deviation of the mode index from the theoretically predicted value. The corrugation of the surface of the dielectric layer can also lead to scattering of the guided mode. Both errors can change the interference pattern of the modes in the MMI section and thereby change the results. For dielectric-loaded plasmonic nanocircuits with a rather thick dielectric layer (> 500 nm), photonic modes are coexisting and the mode index can be significantly affected by the thickness uncertainty. For nanocircuits with a very thin dielectric layer (< 100 nm), the near field of the surface plasmons extends out of the dielectric layer. In this case, the thickness variation can lead to the variation of the “effective surrounding index”, resulting in the index change of the plasmonic modes^32,33^. Therefore, the thickness of the dielectric layer should be carefully controlled.

In conclusion, we have theoretically studied the guided modes in dielectric-loaded plasmonic waveguides. Using the DLP waveguide, we have designed an XNOR logic gate based on MMI and demonstrated that introducing an amplitude modulator at the input port can significantly enhance the on/off ratio of the XNOR logic operation. Together with optimized phase modulators, we have achieved an on/off ratio up to 11.26 dB. Introducing an amplitude modulator to the Ref input port is an effective way to optimize the on/off ratio of plasmonic logic gates based on MMI. This approach is not limited to the demonstrated XNOR gate but generally applicable to various logical gates based on MMI for other logic operations. The unique design flexibility of the amplitude modulator allows for a wide range of applications in optimizing logic operations for nano optics and photonic circuits. The dielectric-loaded nature of the proposed plasmonic nanocircuit also facilitates the fabrication because the structures are realizable for nanoimprint and or nanoprinting methods, which are capable of printing dielectric structures with sub-micrometer features on metallic substrates.

## Methods

### Numerical method

Simulations were performed using commercial software (COMSOL) based on finite element method to obtain the power flow distribution of plasmonic logic gates. The effective indices of the guided modes were obtained by solving eigenvalue problems in the RF module. The dielectric function of Au was taken from previously published data^58^. Transverse magnetic incident waves were used for the excitation of the input ports.

## Discussion

The discussion must not contain subheadings.

## Data Availability

The data that support the findings of this study are available from the corresponding author upon reasonable request.
